# The Role of Gap Junction-Mediated Endothelial Cell–Cell Interaction in the Crosstalk between Inflammation and Blood Coagulation

**DOI:** 10.3390/ijms18112254

**Published:** 2017-10-27

**Authors:** Takayuki Okamoto, Koji Suzuki

**Affiliations:** 1 Department of Pharmacology, Faculty of Medicine, Shimane University, 89-1 Enya-cho, Shimane, Izumo 6938501, Japan; 2 Faculty of Pharmaceutical Science, Suzuka University of Medical Science, 3500-3, Minamitamagaki-cho, Mie, Suzuka 5138679, Japan; suzukiko@suzuka-u.ac.jp

**Keywords:** gap junction, connexin, endothelial cell, inflammation, coagulation, leukocyte adhesion

## Abstract

Endothelial cells (ECs) play a pivotal role in the crosstalk between blood coagulation and inflammation. Endothelial cellular dysfunction underlies the development of vascular inflammatory diseases. Recent studies have revealed that aberrant gap junctions (GJs) and connexin (Cx) hemichannels participate in the progression of cardiovascular diseases such as cardiac infarction, hypertension and atherosclerosis. ECs can communicate with adjacent ECs, vascular smooth muscle cells, leukocytes and platelets via GJs and Cx channels. ECs dynamically regulate the expression of numerous Cxs, as well as GJ functionality, in the context of inflammation. Alterations to either result in various side effects across a wide range of vascular functions. Here, we review the roles of endothelial GJs and Cx channels in vascular inflammation, blood coagulation and leukocyte adhesion. In addition, we discuss the relevant molecular mechanisms that endothelial GJs and Cx channels regulate, both the endothelial functions and mechanical properties of ECs. A better understanding of these processes promises the possibility of pharmacological treatments for vascular pathogenesis.

## 1. Introduction

**Figure 1:**
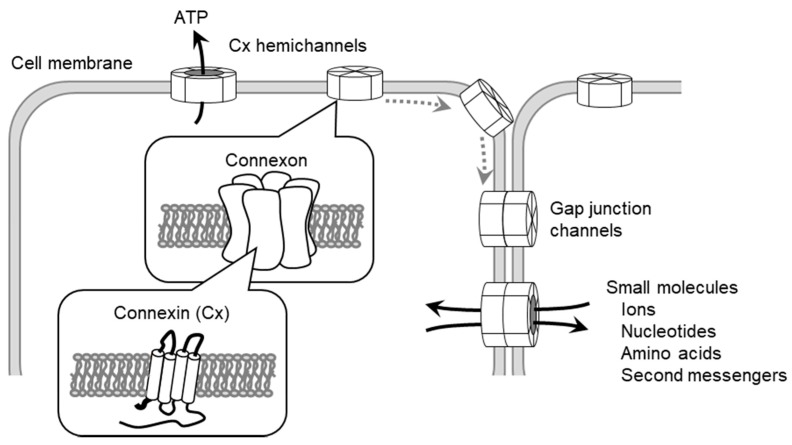
Gap junctions (GJ) and connexin (Cx) hemichannel-mediated intercellular (Cxs are inserted into the cell membrane and assemble into hexameric Cxs or hemichannels. Cx hemichannels transfer intracellular molecules, such as ATP, into extracellular spaces. A connexon pair forms a GJ channel, which transfers intracellular small molecules. ECs communicate with adjacent endothelial cells (ECs), smooth muscle cells (SMCs), leukocytes and platelets and regulate endothelial cellular functions.

Gap junctions (GJs) are comprised of members of the connexin (Cx) protein family [1,2], which contains at least 20 highly conserved proteins in humans and 21 in mice [3,4]. The connexin hexamer forms a hemichannel (connexon) in the plasma membrane and docks to another hemichannel on the adjacent cell (Figure 1) [1,2]. The classification of Cx isoforms is based on their molecular weights, with further designations based on isoform interaction [5]. The Cx has four transmembrane domains and two extracellular loop domains. The amino and carboxyl terminals of Cx proteins are located on the cytoplasmic side of the membrane. The N-terminal domain of Cx26 is likely to work as a plug that affects the closure of GJs [6,7]. The C-terminal domain has several phosphorylation sites that transmit signals in order to control the opening and closing of channels [8]. Phosphorylation of Cx has also been implicated in other biological pathways [9]. The extracellular loops of Cx are responsible for the docking of hemichannels among adjacent cells [1,2]. Although Cx hemichannels consist of homomeric or heteromeric connexins, differences in channel activity and biochemical properties between homomeric and heteromeric channels remain unclear [10]. Most cells express more than one Cx isoform in tissues and various cell type-specific expression patterns [11]. Although the role played by GJs in homocellular interactions is quite well known, their part in heterocellular interactions has only recently been observed.

GJs are channel-like structures that directly connect the cytoplasm of adjacent cells and allow the intercellular movement of small molecules and electron coupling (Figure 1) [1,2]. GJ intercellular communication (GJIC) is essential for the modulation and synchronization of the intracellular environment between adjacent cells. GJIC facilitates this processing by allowing the transfer of intracellular mediators such as ions, amino acids, small metabolites and secondary messengers [12]. In addition, Cx hemichannels are involved in the release of intracellular molecules into extracellular spaces, such as in pannexin channels, resulting in communication links with neighboring cells via the paracrine pathway [13]. Both GJ and Cx hemichannels, therefore, contribute to the maintenance of cellular and tissue functionality.

Vascular component cells, including endothelial cells (ECs), smooth muscle cells (SMCs) and monocytes predominantly express three Cxs: Cx37, Cx40 and Cx43 [14,15]. ECs play a critical role in the regulation of vascular inflammation, blood coagulation [16,17], leukocyte adhesion and extravasation [18], and angiogenesis [19] in response to pro-inflammatory stimuli. Thus, endothelial dysfunction underlies the development of cardiovascular diseases such as atherosclerosis. Vascular component cells dynamically regulate GJ function and Cx expression in response to pro-inflammatory/pro-coagulant stimuli (Table 1). Recent papers have indicated that aberrant GJs and Cx expression in ECs contributes to various endothelial functions including inflammation, blood coagulation and leukocyte adhesion. Below we will discuss the roles of GJs and Cx hemichannels in ECs, which have been implicated in vascular inflammation, blood coagulation and leukocyte adhesion.

## 2. The Patho/Physiological Role of Connexin (Cx) in Progression of Cardiovascular Diseases

Healthy ECs express Cx37 and Cx40, whereas ECs exposed to abnormal turbulent flow and those in microvascular vessels express Cx43 [20]. SMCs of large arteries in vivo predominantly express Cx43 [21,22], while those of some smaller arteries and arterioles express Cx40 [23], and certain types of nonvascular SMCs co-express Cx43 and Cx45 [24,25]. Circulating monocytes express Cx37, whereas monocytes stimulated with pro-inflammatory cytokines [26] and cells in atherosclerotic plaques express Cx43 [27]. In addition, Cx43 is detected in the adventitia, which plays important roles in vessel-wall homeostasis and disease [28]. Adventitia is a perivascular tissue including fibroblasts, microvascular endothelium, nerves, resident macrophages and immune cells [29]. The dysfunction of these Cxs has been implicated as a cause of cardiovascular diseases—for example, cardiac infarction [30], hypertension [31] and atherosclerosis [14,15,26].

Polymorphisms in Cx37 have been associated with myocardial infarction in humans [30], while Cx40 polymorphisms are known to be a risk factor of hypertension [31]. Cx40-deficient mice exhibit highly elevated renin synthesis and secretion from the renin-secreting cells present in the renal juxtaglomerular apparatus, which can result in chronic hypertension [32]. Aberrations in Cx37, Cx40 and Cx43 expression and function contribute to the development of atherosclerosis [14,15]. Cx37^−/−^ ApoE^−/−^ mice develop more aortic lesions than Cx37^+/+^ ApoE^−/−^ mice [26]. In-vivo adoptive transfer experiments have shown that Cx37-deleted monocytes and macrophages enhance recruitment to atherosclerotic lesions, but wild monocyte and macrophages do not do so in enhanced Cx37-deleted endothelium [26]. Cx37 thereby protects against early atherosclerotic lesion formation by negatively regulating monocyte adhesion. In addition, the aortas of Cx37-deficient mice enhance the expression of those pro-inflammatory genes involved in advanced atherosclerosis [33]. Young mice with endothelial-specific deletion of Cx40 develop spontaneous atherosclerotic lesions in their aortas even without a high-cholesterol diet [34]. Moreover, a high-cholesterol diet intensifies the progression of atherosclerosis-associated macrophage infiltration in plaques. The deletion of Cx40 in ECs accelerates the onset of atherosclerosis by promoting leukocyte adhesion. In heterogeneous Cx43 knock-out mice, SMCs not only reduced the expression of Cx43, but also impair its proliferation and differentiation [35]. In this way, Cx43 positively regulates the formation of atherosclerotic lesions. Thus, various influences that are dependent upon Cx expression patterns and cell types have been observed in a wide range of vascular functions associated with the progression of cardiovascular diseases [36].

## 3. The Crosstalk between Inflammation and Blood Coagulation

**Figure 2:**
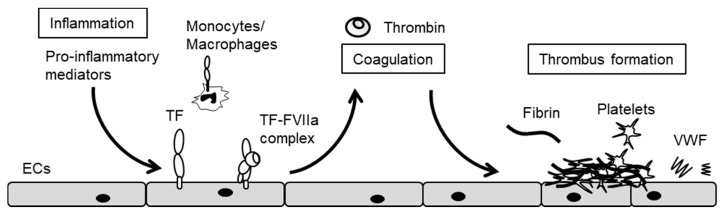
ECs initiate the blood coagulation cascade in response to pro-inflammatory stimuli. Pro-inflammatory mediators induce tissue factor (TF) expression in ECs and initiate a blood coagulation cascade. Thrombin generates fibrin and activates platelets resulting in thrombus formation.

ECs play a pivotal role in the crosstalk between vascular inflammation and blood coagulation, and endothelial dysfunction results from dysregulation of such crosstalk [16,17,37]. In-vitro studies have indicated evidence that pro-inflammatory stimuli induce tissue factor (TF) expression in cultured EC, whereas in-vivo study has not yet confirmed TF expression in the endothelium [38]. This is due to low levels of TF expression on the endothelium, and from TF protein on the endothelium derived from not only endothelium but also cells within the vessel wall, monocyte and extracellular vesicles. Pro-inflammatory stimulation triggers the blood coagulation cascade via TF expression on the surface of ECs (Figure 2) [39]. TF binds to factor VIIa (FVIIa) in serum, thus forming the TF/FVIIa complex that activates FX to FXa. FXa assembles with FVa and forms a pro-thrombinase complex on the surface of ECs. This pro-thrombinase complex then activates prothrombin to thrombin, which in turn converts fibrinogen to fibrin. In addition, pro-inflammatory stimulation induces von Willebrand factor (VWF) release from ECs [40] and anti-fibrinolytic plasminogen activator inhibitor-1 expression in ECs [41], leading to fibrin formation and platelet activation and aggregation on injured endothelium. Thrombin also activates other coagulation factors such as FV, FVIII and FXI, which are components of Xase and prothombinase. In this manner, such complexes rapidly accelerate the generation of thrombin at injured sites, resulting in thrombus formations [42].

**Figure 3:**
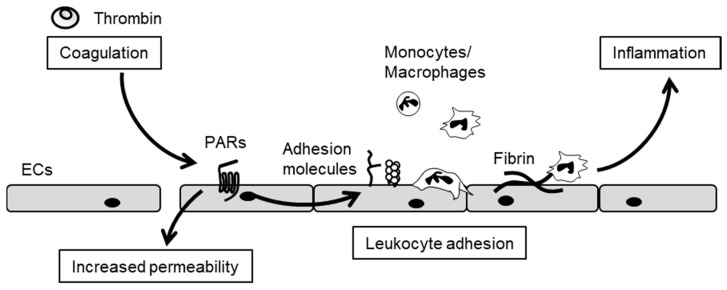
Thrombin induces the inflammatory responses of ECs. Thrombin activates protease-activated receptors (PARs), which induce the disruption of cell–cell junctions and the expression of adhesion molecules, cytokines and chemokines. Thus, the thrombin–PAR pathway contributes to the regulation of vascular permeability, leukocyte adhesion and vascular inflammation.

Conversely, pro-coagulant and anti-coagulant factors such as thrombin, anti-thrombin, thrombomodulin (TM), protein C, fibrinogen and fibrin modulate inflammation. Thrombin, TF/FVIIa complex and FXa exhibit an array of pro-inflammatory activities by cleaving protease-activated receptors (PARs) (Figure 3) [43,44]. Thrombin, therefore, induces leukocyte adhesion molecule expression [43], as well as interleukin-6 (IL-6) and IL-8 production by ECs [45], leading to monocyte and neutrophil chemotaxis [46]. Thrombin also impairs barrier function and increases vascular permeability via the disruption of endothelial cell–cell junctions [47]. A recent study demonstrated that thrombin and fibrin contribute to high-fat diet-induced obesity involved in the induction of chronic inflammation by promoting macrophage recruitment [48]. A direct thrombin inhibitor, dabigatran, protects against the onset of high-fat diet-induced obesity via fibrin-driven inflammation. This is clear evidence that thrombin plays an important role in initiating the inflammation that underlies the pathogenesis of obesity [48]. 

**Figure 4:**
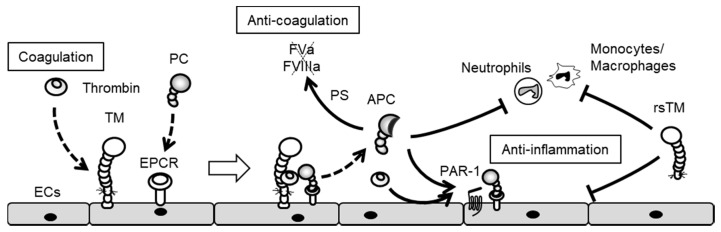
Anti-coagulant and anti-inflammatory effects of the protein C pathway. In a negative feedback loop, the thrombin– thrombomodulin (TM) complex generates activated protein C, which inhibits the blood coagulation cascade by degrading factor Va (FVa) and factor VIIa (FVIIa). In addition, activate protein C (APC) induces anti-inflammatory effects through PAR-1 activation. Both APC and rsTM negatively regulate leukocyte adhesion and EC activation.

Protein C system plays important roles both in anti-coagulant and anti-inflammatory pathways (Figure 4) [17]. While thrombin activates blood coagulation and inflammation under pro-inflammatory conditions, thrombin remains tightly regulated by negative-feedback loops under such physiological conditions. The protein C pathway is initiated by the formation of thrombin and the TM complex on the surface of normal ECs [49]. The interaction of TM with thrombin switches thrombin substrate specificity from fibrinogen to protein C [50]. Protein C then binds to endothelial cell protein C receptor (EPCR) [51], thereby forming a complex with thrombin, TM and EPCR simultaneously on the surface of the endothelium. During the course of this complex formation, protein C is converted to activate protein C (APC) [51]. APC subsequently dissociates itself from the APC–EPCR complex. APC and its cofactor, protein S, dampen the coagulation cascade by degrading both FVa and FVIIIa. In addition to these anti-coagulant effects, APC induces anti-inflammatory and cytoprotective effects by not only activating the PAR-1 [52] and sphingosine 1-phosphate receptor 1 pathways [53] in ECs, but also by inhibiting the transduction of nuclear factor-κB (NF-κB) signaling in monocytes [54,55]. In this PAR-1-derived cytoprotective effect, the activation of PAR-1 co-localizing with EPCR occupied by protein C (PC) or APC is essential, and thereby, thrombin also induces a cytoprotective effect through activation of the EPCR/PAR-1 complex [56]. This suggests that the activation of the EPCR/PAR1 complex is always cytoprotective and not dependent on the protease. Furthermore, APC suppresses lipopolysaccharide (LPS)-induced increases in the pulmonary accumulation of leukocytes, as well as vascular permeability [57]. Moreover, APC, TM and EPCR directly bind to leukocyte integrins, an interaction that may regulate leukocyte adhesion to inflamed endothelium [58,59,60]. Therefore, the protein C pathway plays a pivotal role in the negative regulation of both blood coagulation and inflammation.

## 4. Endothelial Cxs and Inflammation

Within the cells of the blood vessel wall, the expression of Cx37, Cx40 and Cx43 has primarily been observed. The expression of these Cxs is dependent upon vessel type, be it arteries, veins or lymphatic vessels. Typically, Cx37, Cx40 and Cx43 are detected in arterial ECs in the vessels of healthy subjects [15]. In general, Cx37 and Cx40 are co-expressed in ECs [20], whereas Cx43 has been observed in ECs of the microvasculature and at branch points of arteries that experience turbulent blood flow [20]. Cx37 and Cx40 are present in venous ECs, while Cx37, Cx43 and Cx47 are expressed in lymphatic vessels [61]. Both cultured human vein and artery ECs express Cx37, Cx40 and Cx43 in vitro. In addition, we and another group have reported that Cx32 expressed in culture and vessel ECs contributes to both EC–EC communication and hemichannel function [62,63].

Inflammatory stimuli alter Cx expression and GJ function in ECs and other vascular component cells in the context of cardiovascular diseases [64,65]. Pro-inflammatory tumor necrosis factor-α (TNF-α) reduces the expression of Cx37 and Cx40, but not Cx43, in ECs [66]. We have also demonstrated that Cx32 expression is reduced in cultured ECs after exposure to TNF-α for 24 h, although we could not detect significant changes in Cx43 expression [67]. Just as the transcriptional expression of Cxs in ECs weakens, so does TNF-α stimulation (4 h) rapidly attenuate GJ function in cultured ECs, as was evident using a dye-transfer assay [67]. ECs thereby attenuate GJ function in response to TNF-α stimulation without decreasing Cx expression. These results indicate that Cxs are regulated by both transcriptional expression and post-translational modifications. In fact, LPS reduces GJIC between micro-vascular ECs through tyrosine phosphorylation of Cx43 [68,69]. In cultured microvascular ECs from rat and mouse skeletal muscles, LPS not only reduced GJ-mediated electrical coupling along confluent cell monolayers, but also conducted hyperpolarization–depolarization along capillary-like structures. LPS induces tyrosine-phosphorylated Cx43 and serine-dephosphorylated Cx40 [70]. Since LPS-reduced coupling is Cx40- but not Cx43-dependent, only Cx40 dephosphorylation may be consequential [70].

In contrast, SMCs express Cx43 under normal conditions, which increases inflammatory stimulation [71]. The level of Cx43 expression positively correlates with NF-κB activation in human radial arteries in vascular SMCs [72]. While monocytes and macrophages express low levels of Cx37 under normal conditions, treatment with both TNF-α and IFN-γ increases Cx43 expression in monocytes [27]. Foam cells and macrophages in atherosclerotic plaques express both Cx37 and Cx43 [73,74]. Although alterations in Cx expression in response to pro-inflammatory conditions has been observed, tightly regulated GJ function likely stems from the predominantly expressed Cx molecule, depending on the cell type.

Conversely, altered Cx expression and GJ function impact the inflammatory responses of ECs. In particular, the deletion of Cx40 from ECs [34] can promote monocyte adhesion and transmigration, resulting in development of the atherosclerosis that underlies endothelial dysfunction and inflammation. Cx37 knockout mice have exhibited increased expression of pro-inflammatory genes in their aortas [33]. Furthermore, loss-of-function analysis via the use of GJ inhibitors, peptides, antibodies and siRNA has been well documented. We have demonstrated that inhibition of endothelial GJ function by the GJ inhibitors carbenoxolone and oleamide significantly enhanced TNF-α-induced monocyte chemotactic protein-1 (MCP-1) and IL-6 expression by human umbilical vein endothelial cells (HUVECs) [67]. Subsequently, intracellular anti-Cx32 antibody, which blocks GJ formation between ECs via binding to the cytoplasmic domain of Cx32, decreased TNF-α-induced MCP-1 and IL-6 expression in ECs, while overexpression of Cx32 led to a decrease in both of them [67]. Taken together, GJ functionality and predominant Cx expression in ECs may determine the quality and quantity of EC activation in response to inflammatory stimuli.

## 5. Endothelial Cxs and Blood Coagulation

Thrombin and other proteases contribute not only to thrombus formation, but also to EC activation, resulting in the disruption of endothelial barrier function and the induction of vascular inflammation [43,44]. ECs constitutively express the protease-activated receptors PAR-1, PAR-2 and PAR-4, all of which are involved in endothelial functions [75]. Specifically, the thrombin-PAR-1 signal is a major pathway in the regulation of barrier function and vascular inflammation. Although thrombin, as well as cytokines, influences GJ function and Cx expression in ECs, its exact effects remain a subject of controversy. Thrombin attenuates Cx43-based GJIC between fibroblasts via PAR-1 activation [76], which utilizes a Src tyrosine kinase pathway and transiently inhibits Cx43-based GJIC [77]. Moreover, rapid and acute internalization of Cx43-mediated GJ has been observed in primary pulmonary artery ECs following thrombin stimulation via endocytosis [78]. Yet another study has shown that thrombin positively regulates Cx43 expression in ECs and GJ function, processes associated with the disruption of the endothelial barrier [79]. In addition, the GJ inhibitor carbenoxolone and Cx43 knockdown attenuate thrombin-induced vascular permeabilization and phosphorylation of myosin light chain, suggesting that thrombin-induced GJ alterations are involved in the regulation of vascular permeability [79]. Moreover, thrombin promotes ATP release not only from HUVECs through PAR-1 activation [80], but also from lung epithelial cells through the activation of Rho- and Ca^2+^-dependent signaling pathways [81], suggesting that thrombin may induce the opening of Cx hemichannels. Although the effects of thrombin on other endothelial Cxs have not been extensively investigated, these studies indicate the possibility that pro-coagulant thrombin is the modulator of endothelial GJs and Cx hemichannels.

**Figure 5:**
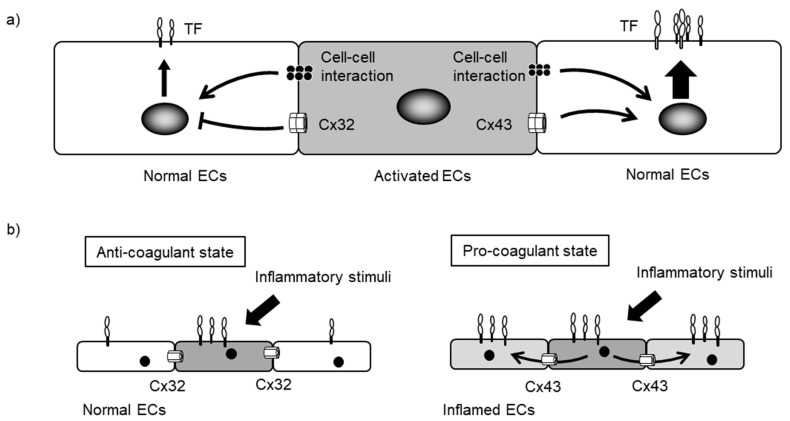
Endothelial Cxs modulate the tissue-factor expression induced by direct cell–cell interaction. (**a**) Activated ECs induce TF expression in adjacent normal ECs via direct contact-based interactions. These direct contact-based pathways help augment blood coagulation at the injured endothelium. Cx32 negatively regulates direct contact-induced TF expression while Cx43 increases it; (**b**) Normal ECs constitutively express Cx32 and induce controlled TF expression while inflamed ECs decrease Cx32 expression and increase Cx43 expression, thereby inducing excessive TF expression. Thus, the predominantly expressed Cxs in ECs may dictate the anti-/pro-coagulant state of endothelial surfaces.

Alteration of endothelial GJ function and Cx expression modulates blood coagulation via TF expression (Figure 5a). We have reported that treatment with non-specific GJ blockers increases TNF-α-induced TF expression [82]. Subsequently, intracellularly transferred anti-Cx32 monoclonal antibody induced TF expression in response to TNF-α stimulation, whereas anti-Cx43 monoclonal antibody reduced TF expression. In the absence of pro-inflammatory stimulation, GJ inhibitors and anti-Cx antibodies exert no influence on TF expression. Therefore, reduced GJ function and Cx expression cannot directly induce TF expression in normal ECs, although they can modulate TF expression in ECs upon pro-inflammatory stimulation, just as occurs with inflammatory cytokine expression. Moreover, the predominant expression of either endothelial Cx32 or Cx43 may dictate pro- or anti-coagulant states on the endothelial surface via TF expression in response to inflammatory stimuli (Figure 5b).

Additionally, we have found that direct cell–cell contacts, including GJs, contribute to TF expression; this was observed by using a co-culture system between TNF-α-activated effector HUVECs and non-treated acceptor HUVECs [82]. Activated ECs induce TF expression in adjacent normal ECs via direct cell–cell contact. The blockade of Cx32 increases direct contact-induced TF expression while blockade of Cx43 reduces it. These results suggest that the establishment of Cx32-mediated GJs provides suppressive signaling in normal ECs, while Cx43 supports inductive signaling in inflamed ECs. Thus, dysfunctional ECs that predominantly express Cx43 may lead to more widespread activation of blood coagulation via increasing TF expression.

Platelets also play an important role in thrombus formation [83]. The expression of Cx32, Cx37, Cx40 and Cx43 have been detected in platelets [84]. GJ structures form between platelets and are involved in intercellular communication within thrombus [84]. Non-specific GJ blockers and specific inhibitors can dampen a range of platelet functions, including platelet aggregation and clot retraction. The inhibition or deletion of Cx37 and Cx40 have reduced not only integrin αIIbβ3-dependent inside-out signaling in platelets, but also granule secretion and platelet activation [84,85]. Additionally, the reduced ATP release from platelets by inhibition of Cx37 and Cx40 suggests that these Cx hemichannel functions may contribute to the regulation of platelet aggregation. In experiments, the bleeding time after tail transection was prolonged in both Cx37- and Cx40-deficient mice. Although inhibition of Cx37 and Cx40 shows effects similar to those of platelets, both Cx37 and Cx40 are able to contribute to platelet regulation in an independent fashion. Another study has demonstrated that deletion of the Cx37 gene in mice shortens bleeding time and increases thrombus propensity [86]. The reasons for this discrepancy remain elusive. Furthermore, the role of other platelet Cxs, such as Cx32 and Cx43, in platelet function remain unclear. Nonetheless, these studies have raised the possibility that platelet Cxs may tightly control the platelet functions underlying thrombus formation and the maintenance of hemostasis.

## 6. Cxs and Leukocyte Adhesion

Leukocyte adhesion to injured ECs and transmigration into sub-endothelial spaces are essential steps of inflammation [87]. ECs regulate leukocyte adhesion and migration by expression of adhesion molecules and chemokines on the surface of cells in response to pro-inflammatory stimuli. Subsequently, circulating monocytes are activated by chemokines. Chemokine signaling elicits an intracellular signaling cascade that eventually induces conformational changes in integrin cytoplasmic domains and then promotes integrin activation. In this way, the integrin present on monocytes achieves a conformational change to a high-affinity active form in the extracellular domain via inside-out signaling. Activated integrins bind their ligand onto the surface of inflamed ECs, thereby permitting monocytes to more-strongly adhere to ECs. The interaction between integrins and integrin ligands contributes not only to adhesion, but also to monocyte migration and transmigration into sub-endothelial spaces. The persistent accumulation of monocytes and macrophages is a hallmark of the atherosclerosis observed in affected vascular lesions [88].

GJs and the Cx hemichannels present in ECs and monocytes have been implicated in monocyte adhesion to endothelium. Cx40- and Cx37-deleted mice experience progressive development of atherosclerotic plaques associated with both monocytes and macrophage recruitment [26,34]. ECs in Cx40-deleted mice increase vascular cell adhesion molecule-1 (VCAM-1) expression, which is a Mac-1 integrin ligand, but decrease 5′-ecto-ATPase CD73 in the aorta [34]. Healthy ECs constitutively express CD73 and degrade extracellular ATP to anti-inflammatory AMP, whereas inflamed ECs decrease CD73 expression and induce inflammatory reactions in response to extracellular ATP [89,90]. Thus, CD73 induces anti-inflammatory signaling via adenosine in order to prevent VCAM-1 expression [34,91]. Endothelial Cx40 promotes CD73 expression, which leads to reduced monocyte adhesion to ECs. The interplay between VCAM-1 and CD73 underlies the mechanism of Cx40-based regulation of leukocyte adhesion. Moreover, extracellular ATP is released from Cx43 hemichannels in ECs in response to thrombin stimulation [80]. Thus, Cx43 hemichannels may positively regulate monocyte adhesion via an ATP pathway.

Cx37-deficient mice exhibit altered global differential gene expression in the aorta towards a pro-inflammatory phenotype, which leads to the development of atherosclerotic plaques [33]. In addition, in-vivo adoptive transfer experiments have shown that Cx37-deleted monocytes and macrophages enhance recruitment to atherosclerotic lesions. In contrast, wild monocytes and macrophages do not enhance their recruitment to Cx37-deleted endothelium. Cx37-deficient monocytes have been shown to adhere more strongly to non-biological surfaces and to EC monolayers. This suggests that Cx37-hemichannels have an anti-adhesive property in relation to monocytes that stems from the release of ATP in the extracellular space; however, the mechanism by which monocyte-released ATP induces this anti-adhesive effect in monocytes is unclear. Further studies will be needed to understand the role played by Cx37 in monocyte adhesion.

**Figure 6:**
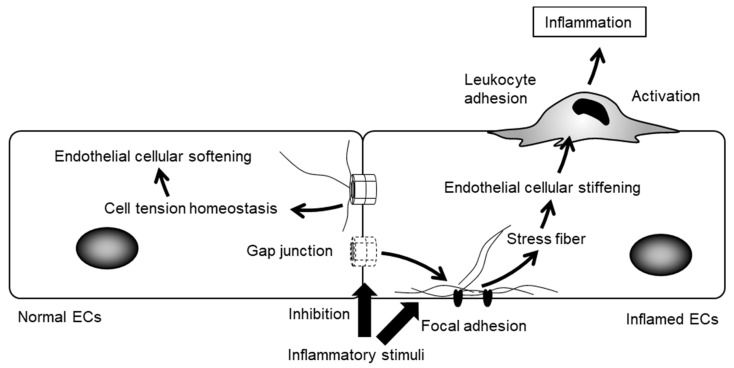
Endothelial GJs modulate cellular stiffness in response to inflammatory stimuli. ECs induce the formation of focal adhesion and stress upon inflammation. Inhibition of endothelial GJs strengthens them, leading to increased cellular stiffness. Leukocytes modulate cell adhesion, spreading and migration, depending on the degree of endothelial cellular stiffness.

It is likely that leukocytes modulate cell adhesion, spreading and migration, depending on the degree of endothelial cellular stiffness (Figure 6) [92,93]. Leukocytes exert durotaxis, a stiffening of the cell-adhesion substrate that is dependent upon cell migration, by mechanically sensing and following gradients of matrix stiffness [94]. In fact, neutrophils increase cell spreading on stiffer ECs, which serve as a substrate for adherent neutrophils [93]. We have recently found that TNF-α stimulation increased endothelial cellular stiffness, a process regulated by cytoskeletal rearrangement and cell–cell interactions [95]. Moreover, blockade of GJs induced cellular stiffening and prolonged TNF-α-induced endothelial cellular stiffening. These results indicate that impaired GJ resulting from inflammation promotes increases in EC stiffness, thereby favoring a microenvironment more suitable for leukocyte adhesion. Thus, leukocytes may sense and respond to endothelial cellular stiffness gradients from the moment of adhesion to the crawling phase of transmigration, including both chemotaxis and durotaxis [96,97]. It has recently been shown that platelets sense the stiffness of the underlying fibrin/fibrinogen substrate, which increases platelet adhesion and spreading on stiffer substrates [98,99]. This raises the possibility that platelets modulate their adhesion depending on endothelial cellular stiffness. Taken together, endothelial cellular stiffening might trigger inflammation and thrombus formation as a consequence of leukocyte and platelet adhesion.

Transmigration of leukocytes across an EC monolayer becomes altered in the presence of Cx-mimetic peptides or gap junction channel blockers [27,100,101]. Although there is some evidence supporting the idea that leukocytes interact with ECs via GJ and Cx hemichannel-mediated communication, heterocellular GJ formation between leukocyte and EC remains a controversial topic. The roles of GJ vis-à-vis leukocytes and ECs in the context of leukocyte adhesion and transmigration have yet to be established.

## 7. The Regulation of Cellular Function by Gap Junctions (GJs) and Cx Channels

Cx channels establish large pores and transfer small molecules to synchronize the intracellular environment with adjacent cells. Metabolome analysis has revealed that over 35,000 molecules could theoretically pass through such channels [13]. Specifically, intracellular second messengers including cAMP, Ca^2+^ and inositol trisphosphate (IP3) pass though Cx channels, and the transfer of these molecules can contribute to the regulation of cellular functions [102,103]. Although it has been suggested that intercellular transfer of Ca^2+^ and ATP release are implicated in vascular inflammation, signaling mediators that pass through GJ and activate inflammation remain unclear. Recent studies have found that some small noncoding RNAs, such as siRNAs and miRNAs, can pass through Cx channels [104,105,106,107]. It is believed that miRNAs must linearize themselves for insertion into these channels, much like a needle. This finding may explain one mechanism by which Cx channels regulate specific gene expression during such cellular functions as pro-/anti-inflammation, pro-/anti-coagulation, cell adhesion, angiogenesis and proliferation. 

GJ- and Cx channel-mediated signaling involves interactions with other protein partners that contribute to not only Cx assembly, trafficking, gating and turnover, but also to the coordinated regulation of cellular functions [10]. Thus, GJIC and signaling are modulated by associating proteins, including regulatory protein phosphatases and protein kinases, catenins, structural proteins and microtubules [108]. For example, Cx32 interacts with Src, calmodulin, claudin, occludin and β catenin [109,110], whereas Cx43 interacts with ZO-1, the Src family, protein kinases, phosphatases and others [111]. It has been suggested that functional differences are determined by proteins’ intracellular domains, in which the C-terminal region of Cx is significantly different. Therefore, GJs and Cx channels may modulate intracellular signaling by using Cx type-dependent protein partners, thereby regulating endothelial cellular functions during vascular inflammation.

In addition, this interaction with associating proteins contributes to the regulation of cellular stiffness [95,112]. The latter has largely been linked to the contractile forces generated by the actomyosin cytoskeleton and filamentous actin [113,114]. Actin bundles bind to α-catenin, which forms a complex with β catenin and vinculin at the cell–cell junction interface. GJs and Cx channels are also thought to interact with a complex consisting of α-catenin, β catenin and cytoskeleton actin [10], much like what occurs with adherence junctions [115]. In addition to cell–cell junctions, cell–substrate interactions involve integrin, which binds the extracellular matrix at the basal membrane, forming a similar complex present in both vinculin and actin filament. Cell–substrate interactions can structurally generate more contractile force than cell–cell interactions. Thus, the balance of adhesive strengths at the integrin-mediated cell–substrate interactions and Cx-mediated cell–cell interactions represents an important mechanism for determining cellular stiffness. This concept is consistent with our results showing that impaired Cx-mediated cell–cell interactions lead to an increase in focal adhesion formation, which is indicative of the augmented cell–substrate interactions associated with cell stiffening [95].

Specifically, both Cx37 and Cx40 have been described to interact with endothelial nitric oxide synthase (eNOS) in aortic ECs [116]. This suggests that both Cx37 and Cx40 may regulate the release of eNOS-derived nitric oxide (NO) [117,118]. The NO generated by healthy endothelium plays an important role in vascular tone and protects against pathological vascular remodeling, hypertension and atherosclerosis [119]. In addition, NO prevents SMC proliferation and migration, leukocyte adhesion and platelet aggregation. In-vitro evidence indicates that Cx37 inhibits eNOS-dependent NO production and release [117]. In agreement with these findings, arteries from Cx37-deficient mice exhibit proper endothelium-dependent relaxation ex vivo. Cx40 also interacts with eNOS, and Cx40-deficient mice are characterized by reduced endothelium-dependent NO-mediated relaxations [118]. Cx40-deficient mice exhibit reduced endothelial Cx37 expression. Thus, it is unclear whether Cx37 and/or Cx40 are responsible for the vascular phenotype of Cx40 deficiency. Cx37 and Cx40 participate in the generation of eNOS-derived NO, thereby contributing to the regulation of endothelial functions.

## 8. Conclusions

The fact that aberrant GJs and Cx channels lead to endothelial dysfunction further emphasizes their importance in the homeostasis of vascular vessels. The endothelial Cxs—Cx32, Cx37, Cx40 and Cx43—have been directly and/or indirectly implicated in a wide range of endothelial cellular functions by mechanisms that have not been fully elucidated. In addition to ECs, SMCs, platelets, monocytes/macrophages and other vascular components, cells express one or more of these Cxs, each representing a different phenotype vis-à-vis their cellular functions. We are beginning to understand how endothelial GJs and Cx channels are linked to the crosstalk and interplay between inflammation and blood coagulation. Further advancements in GJ and Cx research would be obtained from various studies that analyse Cx expression patterns and GJ formation in vascular component cells during vascular inflammation and that elucidate mechanisms governing the regulation of endothelial function and properties. In the future, GJ and Cx hemichannel-mediated cell–cell interaction would be identified as an important system underlying the vascular homeostasis. Further studies should not only provide new insights for the development of pharmacological treatments based on GJs and Cx channels, but also help identify potential targets against vascular diseases.

## Figures and Tables

**Table 1:** Cx expression in vascular component cells and alteration of Cx expression/GJ function upon stimuli.

Cell Type	Cx Expression	Stimuli/Signaling/Process	∆ Cx Expression/GJ Function
Endothelial cell	Cx32, Cx37, Cx40, Cx43	TNF-α	Cx37↑, Cx40↓, Cx32↓, GJ↓
		LPS	Cx40↓, GJ↓
		Thrombin	Cx43↓, GJ↓
		Thrombin	Cx hemichannel↑
Smooth muscle cell	Cx40, Cx43	NF-κB	Cx43↑
Monocyte/Macrophage	Cx37, Cx43	TNF-α and IFN-γ	Cx43↑
		Foam cell	Cx37↑, Cx43↑

## Abbreviations

EC	Endothelial cell
GJ	Gap junction
Cx	Connexin
GJIC	Gap junction intercellular communication
SMC	Smooth muscle cell
TF	Tissue factor
FVIIa	Factor VIIa
VWF	von Willebrand factor
TM	Thrombomodulin
PAR	Protease-activated receptor
IL-6	Interleukin-6
EPCR	Endothelial cell protein C receptor
APC	Activated protein C
NF-κB	Nuclear factor-κB
LPS	Lipopolysaccharide
TNF-α	Tumor necrosis factor-α
MCP-1	Monocyte chemotactic protein-1
HUVEC	Human umbilical vein endothelial cell
VCAM-1	Vascular cell adhesion molecule-1
IP_3_	Inositol trisphosphate
eNOS	Endothelial nitric oxide synthase

## References

[1] Kumar N.M., Gilula N.B. (1996). The gap junction communication channel. Cell.

[2] Saez J.C., Berthoud V.M., Branes M.C., Martinez A.D., Beyer E.C. (2003). Plasma membrane channels formed by connexins: Their regulation and functions. Physiol. Rev..

[3] Willecke K., Eiberger J., Degen J., Eckardt D., Romualdi A., Guldenagel M., Deutsch U., Sohl G. (2002). Structural and functional diversity of connexin genes in the mouse and human genome. Biol. Chem..

[4] Sohl G., Willecke K. (2004). Gap junctions and the connexin protein family. Cardiovasc. Res..

[5] Eastman S.D., Chen T.H., Falk M.M., Mendelson T.C., Iovine M.K. (2006). Phylogenetic analysis of three complete gap junction gene families reveals lineage-specific duplications and highly supported gene classes. Genomics.

[6] Oshima A., Tani K., Hiroaki Y., Fujiyoshi Y., Sosinsky G.E. (2007). Three-dimensional structure of a human connexin26 gap junction channel reveals a plug in the vestibule. Proc. Natl. Acad. Sci. USA.

[7] Oshima A. (2014). Structure and closure of connexin gap junction channels. FEBS Lett..

[8] Yeager M., Nicholson B.J. (1996). Structure of gap junction intercellular channels. Curr. Opin. Struct. Biol..

[9] Hebert C., Stains J.P. (2013). An intact connexin43 is required to enhance signaling and gene expression in osteoblast-like cells. J. Cell. Biochem..

[10] Wei C.J., Xu X., Lo C.W. (2004). Connexins and cell signaling in development and disease. Annu. Rev. Cell. Dev. Biol..

[11] Oyamada M., Oyamada Y., Takamatsu T. (2005). Regulation of connexin expression. Biochim. Biophys. Acta.

[12] Harris A.L. (2007). Connexin channel permeability to cytoplasmic molecules. Prog. Biophys. Mol. Biol..

[13] Esseltine J.L., Laird D.W. (2016). Next-Generation Connexin and Pannexin Cell Biology. Trends Cell. Biol..

[14] Pfenniger A., Chanson M., Kwak B.R. (2013). Connexins in atherosclerosis. Biochim. Biophys. Acta.

[15] Scheckenbach K.E., Crespin S., Kwak B.R., Chanson M. (2011). Connexin channel-dependent signaling pathways in inflammation. J. Vasc. Res..

[16] Esmon C.T. (2005). The interactions between inflammation and coagulation. Br. J. Haematol..

[17] Okamoto T., Tanigami H., Suzuki K., Shimaoka M. (2012). Thrombomodulin: A bifunctional modulator of inflammation and coagulation in sepsis. Crit. Care Res. Pract..

[18] Reglero-Real N., Marcos-Ramiro B., Millan J. (2012). Endothelial membrane reorganization during leukocyte extravasation. Cell. Mol. Life Sci..

[19] Naldini A., Carraro F. (2005). Role of inflammatory mediators in angiogenesis. Curr. Drug Targets Inflamm. Allergy.

[20] Gabriels J.E., Paul D.L. (1998). Connexin43 is highly localized to sites of disturbed flow in rat aortic endothelium but connexin37 and connexin40 are more uniformly distributed. Circ. Res..

[21] Yeh H.I., Dupont E., Coppen S., Rothery S., Severs N.J. (1997). Gap junction localization and connexin expression in cytochemically identified endothelial cells of arterial tissue. J. Histochem. Cytochem..

[22] Ko Y.S., Coppen S.R., Dupont E., Rothery S., Severs N.J. (2001). Regional differentiation of desmin, connexin43, and connexin45 expression patterns in rat aortic smooth muscle. Arterioscler. Thromb. Vasc. Biol..

[23] Little T.L., Beyer E.C., Duling B.R. (1995). Connexin 43 and connexin 40 gap junctional proteins are present in arteriolar smooth muscle and endothelium in vivo. Am. J. Physiol..

[24] Kilarski W.M., Dupont E., Coppen S., Yeh H.I., Vozzi C., Gourdie R.G., Rezapour M., Ulmsten U., Roomans G.M., Severs N.J. (1998). Identification of two further gap-junctional proteins, connexin40 and connexin45, in human myometrial smooth muscle cells at term. Eur. J. Cell. Biol..

[25] Li X., Simard J.M. (1999). Multiple connexins form gap junction channels in rat basilar artery smooth muscle cells. Circ. Res..

[26] Wong C.W., Christen T., Roth I., Chadjichristos C.E., Derouette J.P., Foglia B.F., Chanson M., Goodenough D.A., Kwak B.R. (2006). Connexin37 protects against atherosclerosis by regulating monocyte adhesion. Nat. Med..

[27] Eugenin E.A., Branes M.C., Berman J.W., Saez J.C. (2003). TNF-alpha plus IFN-gamma induce connexin43 expression and formation of gap junctions between human monocytes/macrophages that enhance physiological responses. J. Immunol..

[28] Liao Y., Regan C.P., Manabe I., Owens G.K., Day K.H., Damon D.N., Duling B.R. (2007). Smooth muscle-targeted knockout of connexin43 enhances neointimal formation in response to vascular injury. Arterioscler. Thromb. Vasc. Biol..

[29] Majesky M.W. (2015). Adventitia and perivascular cells. Arterioscler. Thromb. Vasc. Biol..

[30] Yamada Y., Izawa H., Ichihara S., Takatsu F., Ishihara H., Hirayama H., Sone T., Tanaka M., Yokota M. (2002). Prediction of the risk of myocardial infarction from polymorphisms in candidate genes. N. Engl. J. Med..

[31] Firouzi M., Kok B., Spiering W., Busjahn A., Bezzina C.R., Ruijter J.M., Koeleman B.P., Schipper M., Groenewegen W.A., Jongsma H.J., de Leeuw P.W. (2006). Polymorphisms in human connexin40 gene promoter are associated with increased risk of hypertension in men. J. Hypertens..

[32] Wagner C., de Wit C., Kurtz L., Grunberger C., Kurtz A., Schweda F. (2007). Connexin40 is essential for the pressure control of renin synthesis and secretion. Circ. Res..

[33] Derouette J.P., Wong C., Burnier L., Morel S., Sutter E., Galan K., Brisset A.C., Roth I., Chadjichristos C.E., Kwak B.R. (2009). Molecular role of Cx37 in advanced atherosclerosis: A micro-array study. Atherosclerosis.

[34] Chadjichristos C.E., Scheckenbach K.E., van Veen T.A., Richani Sarieddine M.Z., de Wit C., Yang Z., Roth I., Bacchetta M., Viswambharan H., Foglia B. (2010). Endothelial-specific deletion of connexin40 promotes atherosclerosis by increasing CD73-dependent leukocyte adhesion. Circulation.

[35] Kwak B.R., Veillard N., Pelli G., Mulhaupt F., James R.W., Chanson M., Mach F. (2003). Reduced connexin43 expression inhibits atherosclerotic lesion formation in low-density lipoprotein receptor-deficient mice. Circulation.

[36] Billaud M., Lohman A.W., Johnstone S.R., Biwer L.A., Mutchler S., Isakson B.E. (2014). Regulation of cellular communication by signaling microdomains in the blood vessel wall. Pharmacol. Rev..

[37] Levi M., van der Poll T. (2010). Inflammation and coagulation. Crit. Care Med..

[38] Antoniak S., Mackman N. (2016). Editorial Commentary: Tissue factor expression by the endothelium: Coagulation or inflammation?. Trends Cardiovasc. Med..

[39] Mackman N. (2009). The many faces of tissue factor. J. Thromb. Haemost..

[40] Wagner D.D., Bonfanti R. (1991). von Willebrand factor and the endothelium. Mayo Clin. Proc..

[41] Ait-Oufella H., Maury E., Lehoux S., Guidet B., Offenstadt G. (2010). The endothelium: Physiological functions and role in microcirculatory failure during severe sepsis. Intensive Care Med..

[42] Hoffman M., Monroe D.M., Roberts H.R. (1996). Cellular interactions in hemostasis. Haemostasis.

[43] Popovic M., Smiljanic K., Dobutovic B., Syrovets T., Simmet T., Isenovic E.R. (2012). Thrombin and vascular inflammation. Mol. Cell. Biochem..

[44] Coughlin S.R. (2005). Protease-activated receptors in hemostasis, thrombosis and vascular biology. J. Thromb. Haemost..

[45] Johnson K., Choi Y., DeGroot E., Samuels I., Creasey A., Aarden L. (1998). Potential mechanisms for a proinflammatory vascular cytokine response to coagulation activation. J. Immunol..

[46] Bizios R., Lai L., Fenton J.W., Malik A.B. (1986). Thrombin-induced chemotaxis and aggregation of neutrophils. J. Cell. Physiol..

[47] Lum H., Malik A.B. (1996). Mechanisms of increased endothelial permeability. Can. J. Physiol. Pharmacol..

[48] Kopec A.K., Abrahams S.R., Thornton S., Palumbo J.S., Mullins E.S., Divanovic S., Weiler H., Owens A.P., Mackman N., Goss A. (2017). Thrombin promotes diet-induced obesity through fibrin-driven inflammation. J. Clin. Investig..

[49] Conway E.M. (2012). Thrombomodulin and its role in inflammation. Semin. Immunopathol..

[50] Adams T.E., Huntington J.A. (2006). Thrombin-cofactor interactions: Structural insights into regulatory mechanisms. Arterioscler. Thromb. Vasc. Biol..

[51] Stearns-Kurosawa D.J., Kurosawa S., Mollica J.S., Ferrell G.L., Esmon C.T. (1996). The endothelial cell protein C receptor augments protein C activation by the thrombin-thrombomodulin complex. Proc. Natl. Acad. Sci. USA.

[52] Bouwens E.A., Stavenuiter F., Mosnier L.O. (2013). Mechanisms of anticoagulant and cytoprotective actions of the protein C pathway. J. Thromb. Haemost..

[53] Feistritzer C., Riewald M. (2005). Endothelial barrier protection by activated protein C through PAR1-dependent sphingosine 1-phosphate receptor-1 crossactivation. Blood.

[54] Yuksel M., Okajima K., Uchiba M., Horiuchi S., Okabe H. (2002). Activated protein C inhibits lipopolysaccharide-induced tumor necrosis factor-alpha production by inhibiting activation of both nuclear factor-kappa B and activator protein-1 in human monocytes. Thromb. Haemost..

[55] White B., Schmidt M., Murphy C., Livingstone W., O’Toole D., Lawler M., O’Neill L., Kelleher D., Schwarz H.P., Smith O.P. (2000). Activated protein C inhibits lipopolysaccharide-induced nuclear translocation of nuclear factor kappaB (NF-kappaB) and tumour necrosis factor alpha (TNF-alpha) production in the THP-1 monocytic cell line. Br. J. Haematol..

[56] Bae J.S., Rezaie A.R. (2009). Thrombin inhibits nuclear factor kappaB and RhoA pathways in cytokine-stimulated vascular endothelial cells when EPCR is occupied by protein C. Thromb. Haemost..

[57] Murakami K., Okajima K., Uchiba M., Johno M., Nakagaki T., Okabe H., Takatsuki K. (1996). Activated protein C attenuates endotoxin-induced pulmonary vascular injury by inhibiting activated leukocytes in rats. Blood.

[58] Elphick G.F., Sarangi P.P., Hyun Y.M., Hollenbaugh J.A., Ayala A., Biffl W.L., Chung H.L., Rezaie A.R., McGrath J.L., Topham D.J. (2009). Recombinant human activated protein C inhibits integrin-mediated neutrophil migration. Blood.

[59] Kawamoto E., Okamoto T., Takagi Y., Honda G., Suzuki K., Imai H., Shimaoka M. (2016). LFA-1 and Mac-1 integrins bind to the serine/threonine-rich domain of thrombomodulin. Biochem. Biophys. Res. Commun..

[60] Fink K., Busch H.J., Bourgeois N., Schwarz M., Wolf D., Zirlik A., Peter K., Bode C., von Zur Muhlen C. (2013). Mac-1 directly binds to the endothelial protein C-receptor: A link between the protein C anticoagulant pathway and inflammation?. PLoS ONE.

[61] Meens M.J., Kutkut I., Rochemont V., Dubrot J., Kaladji F.R., Sabine A., Lyons O., Hendrikx S., Bernier-Latmani J., Kiefer F. (2017). Cx47 fine-tunes the handling of serum lipids but is dispensable for lymphatic vascular function. PLoS ONE.

[62] Okamoto T., Akiyama M., Takeda M., Gabazza E.C., Hayashi T., Suzuki K. (2009). Connexin32 is expressed in vascular endothelial cells and participates in gap-junction intercellular communication. Biochem. Biophys. Res. Commun..

[63] Thuringer D., Berthenet K., Cronier L., Solary E., Garrido C. (2015). Primary tumor- and metastasis-derived colon cancer cells differently modulate connexin expression and function in human capillary endothelial cells. Oncotarget.

[64] Chadjichristos C.E., Derouette J.P., Kwak B.R. (2006). Connexins in atherosclerosis. Adv. Cardiol..

[65] Severs N.J., Coppen S.R., Dupont E., Yeh H.I., Ko Y.S., Matsushita T. (2004). Gap junction alterations in human cardiac disease. Cardiovasc. Res..

[66] Van Rijen H.V., van Kempen M.J., Postma S., Jongsma H.J. (1998). Tumour necrosis factor alpha alters the expression of connexin43, connexin40, and connexin37 in human umbilical vein endothelial cells. Cytokine.

[67] Okamoto T., Akiyama M., Takeda M., Akita N., Yoshida K., Hayashi T., Suzuki K. (2011). Connexin32 protects against vascular inflammation by modulating inflammatory cytokine expression by endothelial cells. Exp. Cell. Res..

[68] Lidington D., Tyml K., Ouellette Y. (2002). Lipopolysaccharide-induced reductions in cellular coupling correlate with tyrosine phosphorylation of connexin 43. J. Cell. Physiol..

[69] Bolon M.L., Kidder G.M., Simon A.M., Tyml K. (2007). Lipopolysaccharide reduces electrical coupling in microvascular endothelial cells by targeting connexin40 in a tyrosine-, ERK1/2-, PKA-, and PKC-dependent manner. J. Cell. Physiol..

[70] Tyml K. (2011). Role of connexins in microvascular dysfunction during inflammation. Can. J. Physiol. Pharmacol..

[71] Rennick R.E., Connat J.L., Burnstock G., Rothery S., Severs N.J., Green C.R. (1993). Expression of connexin43 gap junctions between cultured vascular smooth muscle cells is dependent upon phenotype. Cell. Tissue Res..

[72] Arishiro K., Hoshiga M., Ishihara T., Kondo K., Hanafusa T. (2010). Connexin 43 expression is associated with vascular activation in human radial artery. Int. J. Cardiol..

[73] Kwak B.R., Mulhaupt F., Veillard N., Gros D.B., Mach F. (2002). Altered pattern of vascular connexin expression in atherosclerotic plaques. Arterioscler. Thromb. Vasc. Biol..

[74] Polacek D., Lal R., Volin M.V., Davies P.F. (1993). Gap junctional communication between vascular cells. Induction of connexin43 messenger RNA in macrophage foam cells of atherosclerotic lesions. Am. J. Pathol..

[75] Nieman M.T. (2016). Protease-activated receptors in hemostasis. Blood.

[76] Van Zeijl L., Ponsioen B., Giepmans B.N., Ariaens A., Postma F.R., Varnai P., Balla T., Divecha N., Jalink K., Moolenaar W.H. (2007). Regulation of connexin43 gap junctional communication by phosphatidylinositol 4,5-bisphosphate. J. Cell. Biol..

[77] Postma F.R., Hengeveld T., Alblas J., Giepmans B.N., Zondag G.C., Jalink K., Moolenaar W.H. (1998). Acute loss of cell-cell communication caused by G protein-coupled receptors: A critical role for c-Src. J. Cell. Biol..

[78] Baker S.M., Kim N., Gumpert A.M., Segretain D., Falk M.M. (2008). Acute internalization of gap junctions in vascular endothelial cells in response to inflammatory mediator-induced G-protein coupled receptor activation. FEBS Lett..

[79] O’Donnell J.J., Birukova A.A., Beyer E.C., Birukov K.G. (2014). Gap junction protein connexin43 exacerbates lung vascular permeability. PLoS ONE.

[80] Godecke S., Roderigo C., Rose C.R., Rauch B.H., Godecke A., Schrader J. (2012). Thrombin-induced ATP release from human umbilical vein endothelial cells. Am. J. Physiol. Cell. Physiol..

[81] Seminario-Vidal L., Kreda S., Jones L., O’Neal W., Trejo J., Boucher R.C., Lazarowski E.R. (2009). Thrombin promotes release of ATP from lung epithelial cells through coordinated activation of rho- and Ca^2+^-dependent signaling pathways. J. Biol. Chem..

[82] Okamoto T., Akita N., Hayashi T., Shimaoka M., Suzuki K. (2014). Endothelial connexin 32 regulates tissue factor expression induced by inflammatory stimulation and direct cell-cell interaction with activated cells. Atherosclerosis.

[83] Vaiyapuri S., Flora G.D., Gibbins J.M. (2015). Gap junctions and connexin hemichannels in the regulation of haemostasis and thrombosis. Biochem. Soc. Trans..

[84] Vaiyapuri S., Jones C.I., Sasikumar P., Moraes L.A., Munger S.J., Wright J.R., Ali M.S., Sage T., Kaiser W.J., Tucker K.L. (2012). Gap junctions and connexin hemichannels underpin hemostasis and thrombosis. Circulation.

[85] Vaiyapuri S., Moraes L.A., Sage T., Ali M.S., Lewis K.R., Mahaut-Smith M.P., Oviedo-Orta E., Simon A.M., Gibbins J.M. (2013). Connexin40 regulates platelet function. Nat. Commun..

[86] Angelillo-Scherrer A., Fontana P., Burnier L., Roth I., Sugamele R., Brisset A., Morel S., Nolli S., Sutter E., Chassot A. (2011). Connexin 37 limits thrombus propensity by downregulating platelet reactivity. Circulation.

[87] Park E.J., Yuki Y., Kiyono H., Shimaoka M. (2015). Structural basis of blocking integrin activation and deactivation for anti-inflammation. J. Biomed. Sci..

[88] Mantovani A., Garlanda C., Locati M. (2009). Macrophage diversity and polarization in atherosclerosis: A question of balance. Arterioscler. Thromb. Vasc. Biol..

[89] Zukowska P., Kutryb-Zajac B., Toczek M., Smolenski R.T., Slominska E.M. (2015). The role of ecto-5′-nucleotidase in endothelial dysfunction and vascular pathologies. Pharmacol. Rep..

[90] Fuentes E., Palomo I. (2015). Extracellular ATP metabolism on vascular endothelial cells: A pathway with pro-thrombotic and anti-thrombotic molecules. Vascul. Pharmacol..

[91] Zernecke A., Bidzhekov K., Ozuyaman B., Fraemohs L., Liehn E.A., Luscher-Firzlaff J.M., Luscher B., Schrader J., Weber C. (2006). CD73/ecto-5′-nucleotidase protects against vascular inflammation and neointima formation. Circulation.

[92] Sun C., Wu M.H., Yuan S.Y. (2011). Nonmuscle myosin light-chain kinase deficiency attenuates atherosclerosis in apolipoprotein E-deficient mice via reduced endothelial barrier dysfunction and monocyte migration. Circulation.

[93] Stroka K.M., Aranda-Espinoza H. (2011). Endothelial cell substrate stiffness influences neutrophil transmigration via myosin light chain kinase-dependent cell contraction. Blood.

[94] Haeger A., Wolf K., Zegers M.M., Friedl P. (2015). Collective cell migration: Guidance principles and hierarchies. Trends Cell. Biol..

[95] Okamoto T., Kawamoto E., Takagi Y., Akita N., Hayashi T., Park E.J., Suzuki K., Shimaoka M. (2017). Gap junction-mediated regulation of endothelial cellular stiffness. Sci. Rep..

[96] Plotnikov S.V., Pasapera A.M., Sabass B., Waterman C.M. (2012). Force fluctuations within focal adhesions mediate ECM-rigidity sensing to guide directed cell migration. Cell.

[97] Huveneers S., Daemen M.J., Hordijk P.L. (2015). Between Rho(k) and a hard place: The relation between vessel wall stiffness, endothelial contractility, and cardiovascular disease. Circ. Res..

[98] Qiu Y., Ciciliano J., Myers D.R., Tran R., Lam W.A. (2015). Platelets and physics: How platelets “feel” and respond to their mechanical microenvironment. Blood Rev..

[99] Qiu Y., Brown A.C., Myers D.R., Sakurai Y., Mannino R.G., Tran R., Ahn B., Hardy E.T., Kee M.F., Kumar S. (2014). Platelet mechanosensing of substrate stiffness during clot formation mediates adhesion, spreading, and activation. Proc. Natl. Acad. Sci. USA.

[100] Oviedo-Orta E., Errington R.J., Evans W.H. (2002). Gap junction intercellular communication during lymphocyte transendothelial migration. Cell. Biol. Int..

[101] Zahler S., Hoffmann A., Gloe T., Pohl U. (2003). Gap-junctional coupling between neutrophils and endothelial cells: A novel modulator of transendothelial migration. J. Leukoc. Biol..

[102] Hernandez V.H., Bortolozzi M., Pertegato V., Beltramello M., Giarin M., Zaccolo M., Pantano S., Mammano F. (2007). Unitary permeability of gap junction channels to second messengers measured by FRET microscopy. Nat. Methods.

[103] Goldberg G.S., Lampe P.D., Nicholson B.J. (1999). Selective transfer of endogenous metabolites through gap junctions composed of different connexins. Nat. Cell. Biol..

[104] Hong X., Sin W.C., Harris A.L., Naus C.C. (2015). Gap junctions modulate glioma invasion by direct transfer of microRNA. Oncotarget.

[105] Kizana E., Cingolani E., Marban E. (2009). Non-cell-autonomous effects of vector-expressed regulatory RNAs in mammalian heart cells. Gene Ther..

[106] Lemcke H., Steinhoff G., David R. (2015). Gap junctional shuttling of miRNA—A novel pathway of intercellular gene regulation and its prospects in clinical application. Cell. Signal..

[107] Brink P.R., Valiunas V., Gordon C., Rosen M.R., Cohen I.S. (2012). Can gap junctions deliver?. Biochim. Biophys. Acta.

[108] Giepmans B.N. (2004). Gap junctions and connexin-interacting proteins. Cardiovasc. Res..

[109] Duffy H.S., Iacobas I., Hotchkiss K., Hirst-Jensen B.J., Bosco A., Dandachi N., Dermietzel R., Sorgen P.L., Spray D.C. (2007). The gap junction protein connexin32 interacts with the Src homology 3/hook domain of discs large homolog 1. J. Biol. Chem..

[110] Talhouk R.S., Mroue R., Mokalled M., Abi-Mosleh L., Nehme R., Ismail A., Khalil A., Zaatari M., El-Sabban M.E. (2008). Heterocellular interaction enhances recruitment of alpha and beta-catenins and ZO-2 into functional gap-junction complexes and induces gap junction-dependant differentiation of mammary epithelial cells. Exp. Cell. Res..

[111] Toyofuku T., Akamatsu Y., Zhang H., Kuzuya T., Tada M., Hori M. (2001). c-Src regulates the interaction between connexin-43 and ZO-1 in cardiac myocytes. J. Biol. Chem..

[112] Stroka K.M., Aranda-Espinoza H. (2011). Effects of Morphology vs. Cell-Cell Interactions on Endothelial Cell Stiffness. Cell. Mol. Bioeng..

[113] Schaefer A., Te Riet J., Ritz K., Hoogenboezem M., Anthony E.C., Mul F.P., de Vries C.J., Daemen M.J., Figdor C.G., van Buul J.D., Hordijk P.L. (2014). Actin-binding proteins differentially regulate endothelial cell stiffness, ICAM-1 function and neutrophil transmigration. J. Cell. Sci..

[114] Schaefer A., Hordijk P.L. (2015). Cell-stiffness-induced mechanosignaling—A key driver of leukocyte transendothelial migration. J. Cell. Sci..

[115] Haidari M., Zhang W., Willerson J.T., Dixon R.A. (2014). Disruption of endothelial adherens junctions by high glucose is mediated by protein kinase C-beta-dependent vascular endothelial cadherin tyrosine phosphorylation. Cardiovasc. Diabetol..

[116] Meens M.J., Alonso F., Le Gal L., Kwak B.R., Haefliger J.A. (2015). Endothelial Connexin37 and Connexin40 participate in basal but not agonist-induced NO release. Cell. Commun. Signal..

[117] Pfenniger A., Derouette J.P., Verma V., Lin X., Foglia B., Coombs W., Roth I., Satta N., Dunoyer-Geindre S., Sorgen P. (2010). Gap junction protein Cx37 interacts with endothelial nitric oxide synthase in endothelial cells. Arterioscler. Thromb. Vasc. Biol..

[118] Alonso F., Boittin F.X., Beny J.L., Haefliger J.A. (2010). Loss of connexin40 is associated with decreased endothelium-dependent relaxations and eNOS levels in the mouse aorta. Am. J. Physiol. Heart Circ. Physiol..

[119] Siragusa M., Fleming I. (2016). The eNOS signalosome and its link to endothelial dysfunction. Pflugers Arch. Eur. J. Physiol..

